# A Forensic Approach to Perioperative Deaths After Non-Cardiac Surgery: A Narrative Review

**DOI:** 10.3390/diagnostics16172692

**Published:** 2026-08-24

**Authors:** Lucia Tattoli, Agnese Accogli, Angelo Montana, Irene Pradelle, Andrea De Gasperi, Margherita Neri

**Affiliations:** 1 Section of Legal Medicine, Department of Sciences of Public Health and Pediatrics, University of Turin, Corso Galileo Galilei 22, 10126 Turin, Italy; agnese.accogli@unito.it; 2 Department of Biomedical Sciences and Public Health, University Polytechnical delle Marche, Via Tronto, 10/a, 60121 Ancona, Italy; a.montana@staff.univpm.it; 3 Section of Legal Medicine, University of Modena e Reggio Emilia, Via del Pozzo, 71, 41124 Modena, Italy; irene.pradelle@unife.it; 4 Anesthesia CCM 2, ASST Grande Ospedale Metropolitano Niguarda, Piazza dell’Ospedale Maggiore 3, 20162 Milan, Italy; dottdega@gmail.com; 5 Section of Legal Medicine, Department of Medical Sciences, University of Ferrara, Via Fossato di Mortara 70, 44121 Ferrara, Italy; margherita.neri@unife.it

**Keywords:** perioperative deaths, non-cardiac surgery (NCS), MACEs, MINS, PMI, forensic investigation

## Abstract

Globally, approximately three hundred million individuals undergo non-cardiac surgery each year. Perioperative mortality results from a complex interplay between patient-related factors and procedural variables, including both surgical and anesthetic aspects. Although cardiac surgery has a well-established risk profile for acute cardiovascular events, major non-cardiac surgery also carries significant—yet often underrecognized—cardiovascular risks. Approximately half of postoperative deaths following non-cardiac procedures are attributable to cardiovascular complications. Surgical and anesthetic stress responses may induce myocardial injury through several pathophysiological mechanisms. However, the absence of a universally accepted definition of perioperative myocardial injury complicates both diagnosis and management. Furthermore, these injuries frequently occur without symptoms, making them clinically silent and often undetected. Consequently, unexpected postoperative deaths may occur and may lead to allegations of medical malpractice. We conducted a narrative review of existing literature on perioperative myocardial injury and its implications for forensic investigation and medico-legal assessment. This paper highlights the importance of a comprehensive forensic evaluation of perioperative deaths, integrating clinical documentation, autopsy findings, histopathological evidence and ancillary investigations to support accurate medico-legal assessment, recognizing that no single element is sufficient to establish the cause of death in all cases. Four illustrative case studies are presented to demonstrate the medico-legal challenges associated with these events. A structured forensic investigation is essential for accurately determining the cause of death and for distinguishing preventable medical errors from unavoidable adverse outcomes within the context of complex perioperative care.

## 1. Introduction

Perioperative mortality following non-cardiac surgery (NCS) remains a significant global health concern. NCS procedures account for the vast majority of operations performed worldwide, with an estimated annual volume of nearly 300 million cases [1,2]. Despite advances in surgical and anesthetic techniques, perioperative mortality continues to be an important public health issue. More than 1% of patients aged 45 years or older undergoing NCS die in hospital or within 30 days after surgery, with severe perioperative cardiac complications as the leading cause of death [3,4].

Although several algorithms have been developed to estimate the risk of perioperative cardiac events in patients undergoing NCS, predicting cardiovascular complications remains difficult. According to AHA 2024 [2], several indices are available for perioperative cardiovascular risk prediction, and in NCS candidates with known cardiovascular disease (CVD), a validated risk-prediction tool can be useful for estimating the risk of perioperative Major Adverse Cardiovascular Events (MACEs) (COR 2a, LOE B-NR). Unfortunately, perioperative treatment strategies/prediction tools are not able to modify perioperative risk, and future studies should address this issue to inform this practice. Functional capacity assessment (in particular, using the DASI Score) and frailty indexes/scores are also reported as important risk indicators, both with COR 2a and LOE B-NR [2,5]. Cardiac-specific biomarkers have therefore been proposed as additional tools to improve perioperative risk stratification [2,5]. However, to date, no studies have been conducted in patients with elevated preoperative biomarkers to recommend management approaches that improve perioperative cardiovascular outcomes. The utility of preoperative biomarkers in low-risk patients has not been evaluated. According to AHA 2024 [2], in patients with known CVD or those aged <65 or <45 years with symptoms suggestive of CVD undergoing elevated-risk NCS, it is reasonable to measure B-type natriuretic peptide (BNP) or N-terminal pro-B-type natriuretic peptide (NT-proBNP) (COR 2a LOE B-NR) and cardiac Troponin (cTn) (COR 2b, LOE B-NR) prior to surgery to inform perioperative risk.

At the same time, there is currently no global consensus regarding the definition of perioperative myocardial injury or the terminology used to describe this condition [6].

In recent years, increasing attention has been paid to perioperative myocardial injury, particularly Myocardial Injury after Non-cardiac Surgery (MINS) [4,6,7,8,9,10]. Large prospective studies have demonstrated that postoperative troponin elevations are relatively common and strongly associated with both short- and long-term mortality [10,11]. Importantly, most events occur without ischemic symptoms or electrocardiographic abnormalities, making them clinically silent and often undetected [12,13].

The growing recognition of perioperative myocardial injury, especially MINS, has significantly changed the understanding of perioperative mortality. Unexpected deaths occurring after apparently low-risk procedures may raise suspicion of medical malpractice. However, the absence of clinical symptoms, the limited pathological findings at autopsy, and inconsistencies in clinical terminology may complicate medico-legal interpretation.

Although perioperative myocardial injury has been extensively studied in the clinical literature, its implications for forensic investigation and medico-legal assessment remain underexplored. This narrative review provides an overview of the current clinical concepts of perioperative myocardial injury and related conditions, highlighting the associated medico-legal challenges through four representative case studies. In this study, we propose a structured approach to the forensic investigation of these complex events.

Close collaboration between clinicians and medico-legal experts is essential to improve our understanding, interpretation, and management of perioperative deaths.

Throughout this review, the term *perioperative* is used as a general descriptor encompassing the entire surgical course. More specific temporal windows (e.g., intraoperative, postoperative, or within 30 postoperative days) are specified when required by established clinical definitions or study endpoints. This definition excludes the conduct of the operation/procedure itself but encompasses the medical care of patients before, during, and after the procedure. Consideration of the whole pathway, from the initial consideration of surgery to full recovery, offers numerous opportunities to improve clinical outcomes and experience of care directly, in relation to the surgery, and in relation to the patient’s overall health [14].

## 2. Materials and Methods

### 2.1. Search Strategy

We conducted a narrative review of the peer-reviewed literature to identify available evidence on perioperative myocardial injury following non-cardiac surgery (NCS), current clinical definitions, preoperative cardiac risk assessment, and the forensic implications of perioperative cardiac events.

A systematic literature search was performed in three electronic databases: PubMed (National Center for Biotechnology Information (NCBI), U.S. National Library of Medicine (NLM), NIH, Bethesda, MD, USA), Scopus (Elsevier, Amsterdam, Netherlands), and Web of Science (Clarivate, Philadelphia, PA, USA). The search was conducted on 27 December 2025 and included articles published up to that date.

The search strategy was developed using a combination of keywords and Boolean operators adapted to the specific syntax of each database. The following search terms were used and combined when appropriate:

- “Perioperative death” OR “postoperative death” OR “perioperative mortality”;
- “Non-cardiac surgery” OR “NCS”;
- “Perioperative myocardial infarction” OR “perioperative myocardial injury” OR “PMI”;
- “Myocardial injury after non-cardiac surgery” OR “MINS”;
- “Major adverse cardiovascular events” OR “MACEs”;
- “Forensic investigation” OR “medico-legal” OR “autopsy” OR “forensic pathology”.

When applicable, controlled vocabulary terms (MeSH for PubMed; Emtree for Scopus) were used to enhance retrieval precision.

### 2.2. Study Selection and Screening Process

All retrieved records were imported into reference management software, and duplicates were removed. Two authors (L.T. and A.A.) independently screened the titles and abstracts of all identified records to assess their potential relevance. Full-text articles were then retrieved and evaluated independently by the same two authors against predefined eligibility criteria.

Studies were considered eligible if they addressed perioperative myocardial injury, infarction, or major adverse cardiac events following non-cardiac surgery; discussed clinical definitions, diagnostic criteria, pathophysiology, or risk stratification; addressed forensic, medico-legal, or autopsy-related aspects of perioperative deaths; were published in peer-reviewed journals; and were written in English.

The following types of publications were excluded: conference abstracts; non-peer-reviewed reports (e.g., editorials, opinion pieces, letters to the editor without original data); and articles not published in English.

Any disagreements between the two reviewers regarding study selection were resolved through discussion and, when necessary, consultation with a third author (M.N.).

### 2.3. Data Extraction and Synthesis

Data from the included studies were extracted by L.T. and A.A. using a standardized data extraction form, which captured information on study design, population, definitions used, diagnostic criteria, incidence data, mortality outcomes, and forensic implications. Given the narrative nature of this review, a formal quality assessment was not performed. Instead, studies were qualitatively assessed based on their relevance to the following key areas: clarification of the terminology used for perioperative myocardial injury (PMI, MINS, MACEs); distinction among different cardiovascular complications following NCS; description of the pathophysiological mechanisms underlying perioperative cardiac events; discussion of the challenges and limitations of postmortem diagnosis; proposal of structured approaches to forensic investigation.

The synthesis was organized thematically, with a focus on integrating clinical knowledge with forensic perspectives.

### 2.4. Selection and Description of Illustrative Cases

The four illustrative cases presented in this review were selected from the institutional archives of the authors’ forensic pathology units. All cases involved perioperative deaths following non-cardiac surgery for which complete forensic autopsy investigations were performed.

Cases were chosen to represent a spectrum of common and challenging medico-legal scenarios, including unexpected death in a patient with undiagnosed cardiac pathology (Case 1), death related to a recognized but potentially preventable perioperative complication (Case 2), multifactorial death requiring careful causal reconstruction (Case 3) and death in a high-risk patient where deviations from the standard of care were considered (Case 4).

These cases were intended not as a consecutive series but as illustrative examples to highlight the practical application of the proposed forensic framework. All case data were anonymized prior to analysis and presentation.

### 2.5. Author Contributions and Review Process

Study selection, data extraction and interpretation of the evidence were performed independently by at least two authors (L.T. and A.A.) to minimize bias and ensure consistency. The final synthesis and the proposed forensic workflow were discussed and refined through collaborative review involving all co-authors, each of whom contributed their respective clinical and forensic expertise.

## 3. Clinical Background

Although major NCS is generally perceived as less hazardous than cardiac surgery, the perioperative period presents a critical window during which adverse events, particularly cardiovascular complications, may occur [3]. Despite improvements in surgical care, including minimally invasive surgical techniques, enhanced recovery pathways and adherence to clinical guidelines, perioperative mortality, Major Adverse Cardiovascular Events (MACEs) and other adverse outcomes remain frequent worldwide [15].

In the large observational VISION (Vascular Events in Noncardiac Surgery Patients Cohort Evaluation) trial, approximately 1.5% of patients aged 45 years or older admitted to hospital after NCS died within 30 days, with myocardial injury representing the most important contributing factor [16]. According to Kulkarni et al. [17], it is common and often asymptomatic. Defined as troponin elevation exceeding the 99th percentile of reference values within 30 days of noncardiac surgery due to ischemia, the estimated incidence is 12–24% and is higher after nonelective procedures. More than 80% of patients present without symptoms of ischemia or electrocardiographic changes, and the condition goes undetected without troponin measurement.

Perioperative mortality reflects the interaction between patient-related factors and surgical stress. Important determinants include age, comorbidities, urgency, type of surgery, and the patient’s global overall physical status as assessed by the American Society of Anesthesiologists (ASA) classification [3]. Current guidelines, therefore, recommend using validated risk calculators that integrate both patient and procedural variables.

Notably, current guidelines recommend against routine preoperative cardiac testing in patients at low cardiac risk undergoing low-risk surgical procedures, as indicated by the 30-day postoperative incidence of deaths or myocardial infarction of <1% [15,18]. However, considering the latter, two key elements should be taken into account.

Among the most commonly used tools are the Revised Cardiac Risk Index (RCRI), the Gupta Myocardial Infarction and Cardiac Arrest (MICA) calculator, and the American College of Surgeons (ACS) National Surgical Quality Improvement Program (NSQIP) surgical risk calculator. These models differ in derivation cohorts, input variables and outcome definitions and often make inconsistent predictions for the same patient [12,19]. Recent studies have shown disagreement rates of nearly 30% in identifying patients classified as being at low perioperative cardiovascular risk according to different preoperative risk prediction models [20].

Another limitation of traditional risk models is that they were originally developed to predict overt myocardial infarction (MI) or death. Universal postoperative screening studies have demonstrated that most perioperative ischemic events are asymptomatic (65%) or consist solely of biomarker elevation without meeting the criteria for MI [12].

As a result, many clinically significant cardiac injuries remain undetected and are not captured by conventional risk prediction tools. For this reason, there is an ongoing effort to validate risk assessment tools in a large, diverse population to provide more accurate estimates of cardiac event rates in a modern cohort. An alternative approach is proposed in the Canadian Cardiovascular Society guidelines [21], addressed by Kulkarni et al. [17], which is recommended for high-risk patients undergoing non-cardiac surgery. It involves routine troponin measurement, immediate postoperative electrocardiography in the recovery room, daily troponin measurement for 48 to 72 h and multidisciplinary management for high-risk patients. For patients undergoing emergency, urgent or semi-urgent surgery, those aged 65 years or older or aged 18–64 years with cardiovascular disease are deemed high-risk. For patients undergoing elective surgery, those aged 65 years or older, 45–64 years with cardiovascular disease, or with a Revised Cardiac Risk Index score of 1 or more are considered high-risk [21]. In the elective setting, physicians should use the preoperative brain natriuretic peptide (BNP) level, when available, to refine risk assessment; postoperative troponin is recommended when the N-terminal prohormone of BNP (NT-proBNP) level is 300 ng/L or higher or BNP level is 92 ng/L or higher.

Functional capacity, typically assessed using metabolic equivalents (METs), is another cornerstone of preoperative cardiac evaluation. A functional capacity below four METs, which corresponds to the inability to climb two flights of stairs, is considered a marker of increased risk. However, contemporary guidelines recognize the limited accuracy of a patient’s subjectivity during interview-based estimates and are now increasingly recommending structured assessment tools, such as the Duke Activity Status Index (DASI), when feasible [19], or the modified DASI (mDASI) [22].

From a medico-legal perspective, the limitations of current predictive models are particularly relevant. Postoperative cardiac death may occur in patients classified as being at low perioperative cardiovascular risk according to preoperative risk prediction models, potentially leading to an erroneous presumption of negligence if the inherent limitations of these tools are not recognized. In every case, there are relevant factors involved in the definition of “ risk”, represented by the patient and their comorbidities; the type, duration and context of surgery; and/or the combination of the two [23].

Future improvements in risk prediction may involve integrating biomarkers, patient and surgical issues and machine learning algorithms [6,23]. Nevertheless, no single test or intervention has yet demonstrated a clear benefit in asymptomatic patients undergoing preoperative screening [15,19].

Cardiovascular complications after NCS occur along a spectrum ranging from asymptomatic biomarker elevations, referred to as “myocardial injury,” to major events such as MI, heart failure, and arrhythmias. However, the terminology used to describe perioperative myocardial injury remains heterogeneous [24], contributing to diagnostic and medico-legal ambiguity.

### 3.1. Definitions

The literature describes several overlapping entities, including Major Adverse Cardiovascular Events (MACEs), perioperative myocardial infarction or injury (PMI) and myocardial injury after non-cardiac surgery (MINS). These entities differ in their definitions, pathophysiology, and diagnostic criteria, yet the terms are often used interchangeably and are not considered separately in guidelines, leading to confusion in clinical and medico-legal contexts (Table 1). Clear differentiation is essential for accurate causal attribution and evaluation of standards of care.

#### 3.1.1. Major Adverse Cardiovascular Events (MACEs)

MACEs represent a composite clinical endpoint commonly used in cardiovascular research to capture major perioperative cardiovascular complications. They are strongly associated with mortality, health resource utilization and long-term disability following surgery [25].

MACEs typically include MI or myocardial injury, stroke, acute heart failure, clinically significant arrhythmias, coronary revascularization procedures and cardiovascular death [26].

However, the exact components of MACEs vary considerably across studies. Some definitions include broader cardiovascular outcomes or all-cause mortality, whereas others restrict the endpoint to cardiovascular death and nonfatal MI. This heterogeneity has been repeatedly criticized for complicating comparisons between studies and limiting the interpretability of systematic reviews and meta-analyses [25,27].

Several patient-related factors are associated with increased perioperative risk of MACEs, including ischemic heart disease, heart failure, cardiomyopathy, significant arrhythmias, chronic kidney disease, diabetes mellitus, pulmonary disease, obesity and anemia [28].

From a pathophysiological perspective, MACEs in NCS result from the interaction of pre-existing cardiac vulnerability and surgery-related physiological stress. Surgical trauma triggers sympathetic activation, systemic inflammation and hypercoagulability, while anesthesia may induce hemodynamic fluctuations. Additional perioperative factors such as tachycardia, hypertension, hypotension, hypoxia, blood losses and anemia may further impair the myocardial oxygen supply–demand balance [26].

#### 3.1.2. Perioperative Myocardial Infarction/Injury (PMI)

Perioperative myocardial injury (PMI) refers to myocardial damage, as evidenced by an increase in a cardiac injury biomarker, such as cardiac troponin, during the perioperative period. However, no universally accepted temporal definition of PMI currently exists, and the terminology and diagnostic framework are not yet uniform across the literature [17,22]. The term may encompass both ischemic and non-ischemic causes, including sepsis, pulmonary embolism, and myocarditis [29].

PMI is frequently used as an umbrella term encompassing both biomarker-defined myocardial injury and perioperative myocardial infarction, which fulfills the Fourth Universal Definition of MI, requiring acute myocardial injury with evidence of acute myocardial ischemia. Unlike classic myocardial infarction, PMI is context-specific (within the perioperative period) and may reflect several mechanisms, including Type 1 MI (plaque rupture), Type 2 MI (supply–demand mismatch) or isolated myocardial injury that does not fulfill MI criteria. Common triggers include tachycardia, hypotension, acute blood loss and anemia, hypoxia, hypertensive crises, sepsis/septic shock and acute heart failure. Among the ischemic causes of PMI, myocardial oxygen supply–demand imbalance is considered the predominant underlying mechanism. Contemporary studies indicate that more than two-thirds of perioperative myocardial infarctions are attributable to a Type 2 mechanism related to myocardial oxygen supply–demand imbalance [24,29].

In the recent clinical literature, PMI is increasingly used to denote any perioperative troponin elevation indicating myocardial injury with or without typical infarction features [11,30,31,32,33].

#### 3.1.3. Myocardial Injury After Non-Cardiac Surgery (MINS)

MINS has gained increasing recognition as a clinically relevant perioperative entity, affecting approximately 20% of patients undergoing major inpatient non-cardiac surgery [4,13,34]. MINS is defined as an acute postoperative elevation of cardiac troponin above the 99th percentile upper reference limit occurring within 30 days after NCS. This elevation is attributed to an ischemic mechanism in the absence of a non-ischemic cause [29].

Because the presence of ischemic symptoms or ECG changes are not required for diagnosis, MINS is frequently asymptomatic and identifiable only through systematic postoperative troponin monitoring.

While PMI refers broadly to any perioperative myocardial injury or infarction, MINS represents a well-defined subset of perioperative myocardial injury that is presumed ischemic [29,35,36]. MINS is a well-defined clinical entity with established prognostic significance. In large prospective studies, MINS has been associated with a 30-day mortality rate of approximately 9.8% and a 1-year mortality rate of approximately 20%. In contrast, the reported mortality rates for PMI are more heterogeneous because they depend on the underlying mechanism of myocardial injury and whether criteria for myocardial infarction are fulfilled [10,11]. Recent guidelines (2022 ESC; 2024 AHA/ACC) [2,19] emphasize the clinical relevance of biomarker-based detection of perioperative myocardial injury (MINS/PMI), even in the absence of symptoms, highlighting a continuum between myocardial injury and infarction. This is particularly important in non-cardiac surgery (NCS) patients due to its strong prognostic value [1,37].

To further clarify the reasons for excluding clinical signs and symptoms of ischemia from the definition of PMI and MINS, it is important to consider that chest pain may be masked by sedation or postoperative analgesia. Furthermore, continuous postoperative ECG monitoring is not routinely performed, contributing to underdiagnosis [13].

Although the prognostic significance of MINS is well-established, optimal management strategies remain uncertain and represent an important gap in the current evidence of PMI or MINS, warranting further investigation [4,10,24].

## 4. Forensic Perspective

Accurate differentiation among MACEs, PMI, and MINS is crucial not only for clinical research but also for the forensic investigation of perioperative deaths.

MACE is primarily a research construct and does not correspond to a specific pathological entity. Although useful for risk stratification and comparative research, MACE does not identify a specific pathological substrate and therefore has limited direct applicability in medico-legal causation analysis.

In contrast, PMI represents objective evidence of myocardial damage occurring during the perioperative period and encompasses a heterogeneous spectrum of conditions [19,29].

MINS indicates ischemic myocardial injury detected through troponin elevation. Because MINS is frequently asymptomatic and may lack specific macroscopic findings at autopsy, its identification often depends on perioperative biomarker monitoring [17]. When such monitoring has not been conducted, forensic experts may face significant diagnostic uncertainty. Moreover, the silent nature of MINS may raise complex issues regarding informed consent, postoperative surveillance, and the attribution of responsibility for adverse outcomes.

This issue has important medico-legal implications. Death occurring in patients considered at low cardiac risk may raise suspicion of malpractice unless the intrinsic limitations of risk prediction models are properly recognized. Common perioperative cardiac risk calculators were originally designed to predict perioperative MACEs or overt myocardial infarction. As a result, these scores perform moderately well for PMI, particularly for symptomatic or infarction-level events, but their sensitivity is quite limited, especially for asymptomatic or Type 2 ischemic events [38].

Traditional clinical risk scores underperform for MINS because many events occur in patients categorized as low- or intermediate-risk by the RCRI tool or who lack classical risk factors [39,40].

Predictive performance improves when clinical risk scores are combined with biomarkers. Among available biomarkers, preoperative cardiac troponin has shown promising predictive value for postoperative myocardial injury, although its routine use remains debated [32,33,41].

Current guidelines recommend postoperative troponin surveillance, primarily in selected patients at increased perioperative cardiovascular risk rather than as part of universal screening [2,19]. However, clinically relevant PMI and MINS may also occur in patients classified as having a low or intermediate estimated perioperative cardiovascular risk, underscoring the limitations of current risk prediction models [39,40].

## 5. Analysis of Illustrative Case Reports

The cases presented below illustrate scenarios commonly encountered in the forensic investigation of perioperative deaths: unexpected fatal events related to undiagnosed disease and rare complications potentially influenced by perioperative management.

***Case Report 1***

A woman in her eighth decade of life underwent elective neurosurgery (C1–C2 arthrodesis with decompression) for cervical instability and myelopathy. Her medical history included hypertension, dyslipidemia and mild pulmonary emphysema. Preoperative evaluation showed a normal electrocardiogram and chest radiography, and the patient was classified as ASA II with preserved functional capacity. The surgical procedure and anesthesia were uneventful. Shortly after extubation, however, the patient developed sudden oxygen desaturation followed by apnea and cardiac arrest. Cardiopulmonary resuscitation was initiated, and return of spontaneous circulation was achieved after prolonged resuscitation. The patient was transferred to the intensive care unit but later suffered a second cardiac arrest and died.

A forensic autopsy was performed due to suspicion of medical liability. Post-mortem examination revealed cardiomegaly (475 g) with abundant epicardial adipose tissue. No surgical complications or acute coronary occlusion were identified. Histological examination of the heart revealed hypertrophy of the cardiomyocytes in the walls of the right and left atria and the septum, with signs of myocardiosclerosis, but above all, a widespread full-thickness adipose replacement of the right atrial wall extending to the epicardial surface.

Now, conditions characterized by a marked disruption of normal cardiac architecture—such as the distribution of adipose tissue within the atrial myocardium and myocardiosclerosis, as observed in the present case—are predictors of arrhythmogenic events due to the resultant cellular uncoupling, which impedes electrical impulse conduction and generates arrhythmias. In this regard, it should be noted that the significant presence of fibro-adipose tissue within the atrial myocardium has been defined in international literature as “atrial dysplasia,” given the histopathological similarities this condition shares with arrhythmogenic right ventricular dysplasia (ARVD). The latter, also known as arrhythmogenic cardiomyopathy, is characterized by the progressive replacement of myocardial muscle fibers by adipose or fibroadipose tissue. From a clinical standpoint, the resulting anatomopathological picture correlates with the occurrence of hyperkinetic arrhythmias, contractile dysfunction, heart failure and an increased risk of sudden death.

Although the literature on histological abnormalities of the right atrium in patients without cardiovascular disease is relatively scarce, cases of isolated right atrial dysplasia have been reported. The process of fibro-adipose infiltration and remodeling of the right atrium appears to be an age-dependent phenomenon, characterized by a higher prevalence in females over 60 years of age with a BMI ≥ 25 and arterial hypertension. Some authors found, at the atrial level, adipose and/or fibro-adipose infiltrates similar to those detected in patients with ARVD in the absence of clear ventricular myocardial abnormalities [42].

Therefore, just as histological abnormalities of the right ventricle can give rise to ventricular arrhythmias (even fatal ones) in ARVD, similarly, right atrial dysplasia, by interfering with the cardiac conduction system, may constitute a histopathological substrate for supraventricular arrhythmias or sudden cardiac death, even in subjects with no apparent history of cardiovascular disease [43,44].

Therefore, in this case, the key initiating factor was likely cardiac arrest of probably arrhythmic origin on a dysplastic substrate of the right atrium, the effects of which, combined with those of secondary hypoperfusion, were found upon histopathological examination. Indeed, recent cardiac necrosis was detected in sections of the anterior wall of the right ventricle (myofiber waviness) and of the interventricular septum (areas of cardiomyocyte waviness with contraction bands). According to the literature [45], myocardial waviness is the earliest microscopic alteration in ischemic cardiomyocytes, characterized by a regional undulating or wavy configuration of the myocardial fibers. This pattern results from the passive stretching of non-contractile, ischemic myocytes by adjacent viable myocardium during the cardiac cycle and is one of the earliest histological indicators of acute myocardial injury.

Based on the timing of histological changes in acute myocardial ischemia, these findings appear to be associated with a very recent ischemic insult.

Death was attributed to sudden cardiorespiratory failure likely related to an arrhythmogenic substrate associated with right atrial dysplasia [46,47].

The conduct of the healthcare professionals was considered appropriate. The neurosurgical procedure was performed in a technically correct manner. Nevertheless, the intervention was complicated by the occurrence of a catastrophic events—acute respiratory failure due to cardiac arrest, probably of arrhythmic origin in the context of unrecognized fat infiltration of heart wall—which was neither preventable nor foreseeable.

This case highlights the importance of detailed histopathological examination in identifying previously undiagnosed cardiac abnormalities that may explain unexpected perioperative death.

***Case Report 2***

A woman in her seventh decade of life underwent elective arthroscopic surgery for a rotator cuff tear. Preoperative investigations, including electrocardiogram, chest radiography, and blood tests, were unremarkable, and the patient was classified as ASA II. The procedure was performed under general anesthesia combined with an ultrasound-guided brachial plexus block in the beach-chair position (BCP), with the head elevated above an ideal horizontal plane to varying degrees, depending on surgical requirements (20–45° up to 70°, in some cases even 80°) for better surgical access to the shoulder region (improved joint mobility and less blood in the surgical field). The BCP, however, requires extreme attention from an anesthesiologic perspective and specific management and monitoring precautions due to the significant associated challenges, primarily related to cerebral oxygenation and perfusion, which are the reason for this case’s considerable clinical and medicolegal relevance. Even if extremely rare (1.2%, range of 0.2–1.7%, with mortality close to 0.05%), devastating neurological complications can occur (0.07%) including post-anoxic coma, vegetative state and vision loss.

The surgical procedure was completed without apparent complications. However, the patient failed to regain consciousness in the postoperative period and was transferred to the intensive care unit. Diagnostic investigations revealed severe hypoxic–ischemic brain injury, and the patient remained in a persistent vegetative state until death approximately one month later.

A forensic autopsy revealed diffuse cerebral edema, pulmonary edema, contraction band necrosis in the myocardium, and minimal evidence of fat embolism. Multidisciplinary analysis of the clinical records and autopsy findings suggested a hypoxic–ischemic brain injury. The effects of the sitting position on cerebral perfusion and oxygenation during general anesthesia are complex and not yet fully understood. In an awake patient, cerebral perfusion in the sitting position relies on compensatory mechanisms (cerebral autoregulation; the mechanisms are believed to maintain constant cerebral blood flow with a MAP between 50 and 150 mm Hg) based on the orthosympathetic response. Since general anesthesia significantly impairs orthosympathetic activation, hypotension may be common, resulting in cerebral hypoperfusion due to reduced cerebral blood flow, reduced intracranial venous drainage and reduced cerebral oxygenation. Hypertensive and elderly patients may require MAP values of not 50, but at least 70 mm Hg to maintain autoregulation to prevent cerebral ischemia in the sitting position. In the absence of definitive guidelines, a MAP > 70 mm Hg is strongly supported as a target for controlled hypotension (frequently required by the surgeon) and should be carefully considered and planned for on a case-by-case basis before surgery. Patient risk factors and similar information should be included in the consent form, along with the position, type of technique, risks, benefits and the type of cardiovascular, and respiratory and cerebral monitoring to be used. The recommendations for measuring arterial blood pressure are extremely relevant. In patients at risk, invasive arterial pressure should be seriously considered and measured at the “cerebral” level, with the transducer zeroed at the level of the external auditory meatus (tragus). If non-invasive arterial blood pressure (NIBP) is chosen, the “measured” MAP should be corrected to account for the pressure difference “between the cuff and the brain” (auditory meatus, as noted above). In fact, for every 1.25 cm difference between the cuff (usually on the arm rather than at heart level) and the external auditory meatus, the MAP is 1 mm Hg lower at the cerebral level. In BCP, with the external auditory meatus being on average 30 cm above the cuff, the MAP might be close to 47 mm Hg, with a cuff MAP of 70 mm Hg. In this case, it is recommended to keep the MAP within 20% of the preoperative resting baseline and ≥70 mm Hg. Episodes of cerebral desaturation are associated and frequent if tracked. EtCO2 must also be monitored to keep it within normal limits [47,48]. The most probable explanation for the devastating complications that occurred in this case is suboptimal intraoperative blood pressure management and prolonged brain hypoperfusion. No information about the site of the NIBP measurement was available. As noted above, this dramatic event is extremely rare and difficult to predict, but there are clear suggestions for ad hoc intraoperative blood pressure monitoring to help prevent the complication.

This case underscores the importance of integrating perioperative clinical data and pathological findings to understand the origin of a rare but devastating perioperative complication, identify potential deviations from basic safety measures, and guide intraoperative anesthesiologic management and monitoring [48,49,50,51,52].

***Case Report 3***

A man in his eighth decade of life underwent elective day surgery for inguinal hernia repair with a mesh plug. His medical history included diabetes, hypercholesterolemia, a stable abdominal aortic aneurysm, prior transient ischemic attack, patent foramen ovale and right carotid artery occlusion. During the preoperative anesthetic assessment, dual antiplatelet therapy was appropriately discontinued 5 days before surgery. The ECG was normal, but no other cardiac instrumental exams were performed, such as DASI or METs. The procedure was performed under local anesthesia and was completed without complications. The patient was discharged shortly afterward. Approximately 7 h later, he was admitted to the emergency department with vomiting and hypotension. Laboratory tests revealed anemia, and an abdominal CT scan demonstrated a retroperitoneal hematoma with active bleeding from the inferior epigastric artery. At the end of the imaging examination, the patient complained of chest pain, and electrocardiography showed ischemic changes. He was transferred to a tertiary (Hub) center, where he underwent embolization and was subsequently admitted to the Coronary Care Unit for management of an NSTEMI. During hospitalization, he developed hyperkinetic delirium, systolic ventricular dysfunction and atrial fibrillation. On the fourth postoperative day, he suffered an irreversible cardiac arrest despite prolonged resuscitation efforts.

A forensic autopsy revealed cardiomegaly with abundant subepicardial adipose tissue. The heart weighed 586 g, and 80% stenosis of the left anterior descending coronary artery and 85% stenosis of the mid-segment of the right coronary artery were observed. Histopathological examination showed diffuse myocardial fibrosis with necrotic foci, considered extensive myocardial necrosis in a diabetic patient with advanced vascular disease. Death was attributed to an acute chain of events starting from anemia secondary to acute blood loss followed by a discrepant myocardial ischemia in a patient with severe CAD, resulting in NSTEMI and acute cardiac failure.

This case emphasizes the importance of integrating clinical documentation with pathological findings and the critical role of previously undiagnosed cardiac disease in determining death despite appropriate clinical management and excluding a causal contribution of the surgical procedure.

***Case Report 4***

A man in his seventh decade of life underwent elective total hip replacement surgery. The patient had a history of hypertension, morbid obesity, and persistent atrial fibrillation requiring anticoagulant therapy and dilated cardiomyopathy, which were compounded by a recent history of percutaneous coronary intervention. Three drug-eluting stents had been placed less than six months prior, and antiplatelet therapy was still ongoing. A preoperative cardiology assessment identified a significantly high cardiac risk. It also highlighted the need for careful timing when switching from anticoagulant agents to heparin and the importance of measuring troponin levels preoperatively and at 24 and 48 h postoperatively. The surgical procedure was performed, and the patient was admitted directly to the orthopedic ward without an observation period in the recovery room. No continuous ECG monitoring was conducted in the ward, and the patient suffered a cardiac arrest two hours later. Forensic autopsy complete with macroscopic and microscopic findings, aided by immunohistochemical markers, revealed acute myocardial ischemia that occurred approximately three to four hours before death. This suggested it took place either during or shortly after surgery.

This case underscores the critical importance of accurate preoperative risk assessment and appropriate timing of non-urgent surgical intervention (no need for an “urgent” hip replacement), thorough discussion of the risk–benefit ratio and careful evaluation of surgical indications. The absence of these crucial steps in the treatment process was highlighted following a thorough multidisciplinary forensic investigation.

## 6. Discussion

While the clinical aspects of perioperative myocardial injury have been extensively described in the literature, their implications for forensic investigation and medico-legal assessment remain less clearly defined.

The medico-legal expert must reconstruct the causal chain leading to death, distinguish unavoidable complications from preventable adverse events, and determine whether deviations from accepted standards of care contributed to the fatal outcome. This task is particularly challenging because perioperative mortality is often multifactorial. Pathognomonic autopsy findings are frequently absent, and evolving clinical definitions have further complicated interpretation.

The increasing recognition of MINS has profoundly changed the understanding of perioperative mortality. Some deaths previously considered unexplained may in fact represent undetected myocardial injury occurring during the perioperative period [53]. Because MINS is frequently asymptomatic and may not produce macroscopic autopsy findings, forensic experts may face a diagnostic gap when perioperative troponin monitoring was not performed [54,55]. This uncertainty may lead to disputes regarding causation and the standard of care. In this context, forensic investigation must rely on careful integration of clinical documentation, perioperative risk assessment, involvement of a specialist expert in the specific field, autopsy findings and ancillary investigations.

### 6.1. Proposed Forensic Approach to Perioperative Deaths

The forensic investigation of perioperative deaths in NCS should integrate current clinical knowledge with a structured forensic approach (Table 2).

#### 6.1.1. Clinical Reconstruction

The investigation should begin with a detailed analysis of clinical documentation, including preoperative evaluation, comorbidities, surgical indications, anesthesiologic records, intraoperative monitoring and postoperative course.

Attention should be paid to pre-existing comorbidities such as ischemic heart disease, heart failure, chronic kidney disease and frailty as these conditions significantly influence perioperative risk and may represent independent or contributing causes of death. Appropriate and complete informed consent is crucial in this setting.

Understanding surgical technique is crucial for recognizing potential injuries. Forensic pathologists require familiarity with surgical procedures to accurately identify and document injuries as cases may go unnoticed when pathologists lack knowledge of surgical techniques. A multidisciplinary approach, including input from surgeons and anesthesiologists, may help the pathologist assess the correctness of the surgical procedure [56].

It is essential to contextualize the death within the procedure’s expected risk profile. NCS procedures are associated with a well-documented incidence of cardiovascular, respiratory and neurological complications, including perioperative myocardial injury, arrhythmias, thromboembolic events, hemorrhage, sepsis and respiratory failure.

#### 6.1.2. Analysis of the Perioperative Timeline

The temporal relationship between surgery and death must be reconstructed as must the timing, type (BLSD, ALS, ACLS, ECLS), and quality of cardiopulmonary resuscitation.

Iatrogenic complications constitute a significant proportion of perioperative deaths, and each surgical approach and site has its own unique potential complications. A recent retrospective study identified a rate of iatrogenic perioperative deaths of 24.4%, with about one-third of deaths occurring after the first postoperative day [57]. Patients undergoing multiple interventions have a significantly higher rate of iatrogenic deaths (35.6% vs. 16.6% for single procedures). Elective procedures show unexpectedly greater iatrogenic death rates (33.3% vs. 13.2% for emergencies) [57].

Cardiac arrest during induction often reflects anesthetic factors (64% associated with airway management complications), and intraoperative arrests may relate to surgical events, whereas postoperative arrests frequently involve hemorrhagic, respiratory or metabolic complications [58].

As highlighted by Vardhan Reddy et al., [59] significant discrepancies between clinical diagnoses and autopsy findings are observed in up to 47% of cases, particularly in the case of hemorrhage, sepsis, pulmonary embolism, and anastomotic leaks. These findings underscore the irreplaceable role of autopsy not only in forensic practice but also as a tool for enhancing care and evidence-based practice, both of which remain underused [59].

Assessment of preventability considers whether timely recognition and intervention could have prevented death. Failure to identify the lesion constitutes a specific medical error or deviation from standard care. Conversely, some deaths are unpreventable despite appropriate, documented care.

#### 6.1.3. Autopsy Examination

Autopsy remains a cornerstone of forensic investigation in perioperative deaths. The best approach can vary depending on the surgical procedure and complications. A complete autopsy should include careful examination of the cardiovascular system, lungs, central nervous system and surgical site, with documentation of any evidence of hemorrhage, thromboembolism, infection or technical complications.

After death, all surgical and anesthetic devices should remain in place to guarantee correct placement and patency checks, with extensive photographic documentation. Pre-dissection imaging can help assess anatomical positioning when clinical data cast doubt on correct positioning [56].

A thorough autopsy requires knowledge of any prior surgical approach to identify potential anatomical variations or the presence of devices since once anatomical structures, wires/lines and electrodes are cut, they are no longer evaluable [56]. A distinction between injuries caused by treatment or resuscitation and injuries causing death should be assessed.

Microscopic examination provides essential diagnostic information beyond gross findings. Current guidelines recommend histopathological examination of major organs, including the heart, lungs, liver and kidneys, to identify potential causes of death, with specific sampling guidelines for cardiac and neuropathological assessment [56]. Histological examination is crucial, especially for detecting early myocardial ischemia, microinfarctions or stress-related myocardial injury that may not be macroscopically evident. However, the forensic pathologist must also be aware of the temporal limitations of histopathology as perioperative myocardial injury may occur shortly before death and remain histologically silent. Likewise, contraction-band necrosis should be interpreted with caution because it is not specific for ischemic myocardial injury and may occur following catecholamine excess or cardiopulmonary resuscitation [60]. Similarly, although immunohistochemistry may provide useful supportive evidence for the detection of early myocardial ischemia, these findings should be interpreted in conjunction with the overall clinical and pathological context and should not be considered diagnostic in isolation [61]. Therefore, evidence of myocardial ischemia or myocardial injury should not automatically be considered proof that myocardial injury was the immediate cause of death, which would require the integration of all available clinical information, perioperative findings, autopsy results, histopathology and ancillary investigations. In this context, post-mortem biochemistry—such as cardiac troponins—may provide supportive information. However, interpretation must be approached with caution due to post-mortem changes and the lack of standardized reference values [55,56,62].

#### 6.1.4. Ancillary Investigations

Additional analyses may include toxicology, microbiology, post-mortem imaging and, in selected cases, genetic testing for inherited cardiac disorders [56,63,64].

Toxicological analyses are essential to assessing anesthetic agents, like opioids, sedatives, neuromuscular blockers and other perioperative drugs, to exclude overdose, drug interactions, or inadequate reversal. Interpretation of plasma drug concentrations must be cautious as they are easily altered by postmortem changes and perimortem rescue interventions, like high-volume fluid infusion in the case of hypotension, which can lead to hemodilution [65].

In selected cases, microbiological studies may be indicated to evaluate suspected sepsis or surgical site infections [63].

Since biochemical, toxicological and microbiological samples taken during dissection may be affected by postmortem changes and contamination, samples obtained during life are potentially useful. Given that such samples are not commonly kept for long in laboratories, the pathologist must take prompt action to ensure they are retained [63].

Post-mortem imaging enhances investigation capabilities and aids in determining the cause of death. Current guidelines recommend both postmortem computed tomography and magnetic resonance imaging as adjuncts to traditional autopsy. Postmortem imaging supports collaborative approaches with clinical teams [66,67].

#### 6.1.5. Causation Assessment

The final step requires integrating all available findings to determine whether death resulted from natural disease, an unavoidable complication, or a preventable adverse event.

In a medico-legal analysis, causation is assessed probabilistically rather than deterministically. Experts must evaluate whether any deviation from standard care made a substantial causal contribution to death while considering the inherent risks of surgery and the patient’s baseline condition. This involves differentiating among the consequences of the natural progression of the disease, recognized complications, and preventable adverse events.

The quality of medical documentation is central to this analysis. Incomplete or inconsistent records may themselves constitute a medico-legal issue as they hinder the reconstruction of events and may suggest deficiencies in clinical management.

Deviation from the standard of care may include inadequate pre-operative assessment, inappropriate patient selection, technical surgical errors, anesthetic mismanagement, including intraoperative monitoring, delayed recognition of complications and insufficient post-operative monitoring. Conversely, the presence of a known and unavoidable complication does not automatically exclude liability if risk-mitigation strategies were insufficient or poorly documented.

From a legal standpoint, the evolving understanding of perioperative complications underscores the dynamic nature of standards of care. Medico-legal assessments must therefore be grounded in contemporary scientific knowledge and the guidelines available at the time of the event, avoiding retrospective bias.

In a retrospective review by Tabib et al. [68] of 1700 forensic autopsies conducted for unexpected sudden cardiac death, 50 fatalities were identified as potentially associated with surgery and/or anesthesia. The patients involved were young and had no prior history of cardiac disease. All patients were classified as ASA I, and the surgical procedures were of low risk. Cardiac arrest occurred during the induction of anesthesia in 16% of cases, during surgery in 64% and at the end of surgery in 20%. Investigations and expert reports requested by the public prosecutor did not reveal any of the common causes of death typically linked to surgical or anesthetic complications. However, pathological examination detected cardiac abnormalities in 47 cases, including arrhythmogenic right ventricular cardiomyopathy (18 cases), silent coronary artery disease (10 cases), cardiomyopathy (8 cases), structural abnormalities of the His bundle (9 cases), mitral valve prolapse (1 case) and acute myocarditis (1 case). Once again, these findings show how autopsy, which enables the correct identification of the underlying cause of death in patients classified as having a low estimated perioperative cardiovascular risk, can not only provide an answer in cases of malpractice litigation but also make a substantial contribution to improving preoperative risk evaluation and evidence-based practice [68].

Perioperative deaths frequently result in significant emotional distress for families and healthcare providers and often lead to legal disputes. The forensic expert must maintain scientific objectivity, involve recognized experts in the specific setting, and address ethical issues related to transparency, informed consent, and risk communication. In particular, the adequacy of preoperative informed consent should be evaluated to ensure that the patient was properly informed of the specific risks associated with noncardiac surgery and of the patient’s individual risk profile.

The increasing complexity of perioperative deaths investigation extends beyond clinical practice and has important implications for medico-legal assessment. From a scientific perspective, the findings of this review support a multidisciplinary approach integrating forensic pathology with contemporary perioperative cardiovascular medicine to achieve accurate causal attribution. Within the Italian legal framework, multidisciplinary expert consultation involving both medico-legal and relevant clinical specialists represents an established component of malpractice litigation (Law No. 24 of 8 March 2017 “Provisions on Patient Safety and the Safety of Healthcare, as Well as the Professional Liability of Healthcare Professionals”, Official Gazette of the Italian Republic, 17 March 2017) [69].

Comparable principles are reflected in professional guidance from other jurisdictions. For example, the Academy of Medical Royal Colleges in the United Kingdom recommends that healthcare professionals acting as expert witnesses maintain up-to-date clinical expertise, work within their field of competence and provide opinions consistent with current scientific evidence. Similarly, the French National Academy of Medicine has emphasized the importance of specialty-specific expertise and multidisciplinary expert assessment in medically complex liability cases [70,71].

## 7. Conclusions

Perioperative deaths following NCS presents a complex clinical and medico-legal challenge. The recognition of perioperative myocardial injury, particularly MINS, has substantially reshaped the interpretation of postoperative mortality. However, the frequent absence of symptoms and the limited pathological evidence at autopsy continue to pose significant challenges for forensic investigation [55,63].

This review highlights the importance of a multidisciplinary approach integrating forensic pathology with contemporary perioperative medicine. Accurate causal attribution requires integrating clinical documentation, perioperative risk assessment, biomarker data, autopsy findings, histopathology and ancillary investigations. Such an integrated approach may improve cause-of-death determination, reduce diagnostic uncertainty and ultimately contribute to patient safety and healthcare quality.

## Figures and Tables

**Table 1:** Comparative overview of Major Adverse Cardiovascular Events (MACEs), Perioperative Myocardial Infarction/injury (PMI), and Myocardial Injury after Non-cardiac Surgery (MINS).

Term	Description	Core Criteria	Clinical Meaning	
MACEs	Composite outcome measure	MI, cardiovascular death, stroke (±others)	Variable composite endpoint not a diagnosis	Research Endpoint
PMI	Perioperative myocardial injury/infarction	Postoperative troponin elevation ±ischemic features	Broad, heterogeneous concept	Diagnosis
MINS	Ischemic myocardial injury after non-cardiac surgery	Troponin elevation > 99th percentile, ischemic cause presumed, no non-ischemic explanation	Specific, prognostically relevant entity	Clinical Syndrome

**Table 2:** Forensic investigation workflow in perioperative deaths after non-cardiac surgery.

Step	Key Actions	Diagnostic Focus	Medico-Legal Relevance
Clinical reconstruction	Review clinical records; assess comorbidities; analyze surgical indication and technique; multidisciplinary evaluation	Baseline risk; perioperative complications (cardiac, hemorrhagic, thromboembolic, infectious)	Defines risk profile; identifies alternative causes; evaluates appropriateness of care
Perioperative timeline analysis	Reconstruct perioperative phases; identify complications; assess timing of events; evaluate preventability	Anesthesiologic vs. surgical vs. postoperative causes; timing of cardiac arrest; rhythm of presentation if possible and if it is defibrillable or not	Identifies errors or delays; distinguishes preventable vs. unavoidable events
Autopsy examination	Full macroscopic exam; evaluate surgical site and devices; histopathology; post-mortem biochemistry	Hemorrhage, thrombosis, infection, myocardial injury (including subclinical)	Provides objective cause of death; detects missed diagnoses; validates clinical findings
Ancillary investigations	Toxicology; microbiology; post-mortem imaging; genetic testing (selected cases); review ante-mortem samples	Drug effects; sepsis; occult pathology; inherited cardiac disease	Improves diagnostic accuracy; clarifies uncertain cases
Causation assessment	Integrate all findings; classify death; evaluate deviations from standard care; assess causal contribution	Natural disease vs. complication vs. preventable adverse events	Establishes causation; supports liability assessment
Medico-legal considerations	Evaluate documentation; informed consent; adherence to guidelines, and avoid hindsight bias	Documentation gaps; communication; ethical issues	Ensures robust, defensible expert opinion

## Data Availability

No new data were created or analyzed in this study. The cases presented are completely anonymized and only served an explanatory purpose. Data sharing is not applicable to this article.

## Abbreviations

The following abbreviations are used in this manuscript:

ACS	American College of Surgeons
ASA	American Society of Anesthesiologists
BCP	Beach-chair position
MACE	Major Adverse Cardiovascular Events
METs	Metabolic equivalents
MI	Myocardial infarction
MICA	Myocardial Infarction and Cardiac Arrest
MINS	Myocardial Injury after Non-cardiac Surgery
NCS	Non-cardiac surgery
NSQIP	National Surgical Quality Improvement Program
PMI	Perioperative myocardial infarction or injury
RCRI	Revised Cardiac Risk Index
